# Farm Animals Are Long Away from Natural Behavior: Open Questions and Operative Consequences on Animal Welfare

**DOI:** 10.3390/ani11030724

**Published:** 2021-03-06

**Authors:** Alberto Cesarani, Giuseppe Pulina

**Affiliations:** 1 Animal and Dairy Science Department, University of Georgia, Athens, GA 30602, USA; 2 Department of Agraria, University of Sassari, 07100 Sassari, Italy; gpulina@uniss.it

**Keywords:** bioethics, domestication, genetic selection, animal behavior, animal welfare

## Abstract

**Simple Summary:**

Animal welfare is a very important issue. One of the tasks of researchers is to provide explanations and possible solutions to questions arising from non-experts. This work analyzes part of the extensive literature on relationships between selection and domestic, mainly farm, animals’ behavior and deals with some very important themes, such as the role of regulations, domestication, and selection.

**Abstract:**

The concept of welfare applied to farm animals has undergone a remarkable evolution. The growing awareness of citizens pushes farmers to guarantee the highest possible level of welfare to their animals. New perspectives could be opened for animal welfare reasoning around the concept of domestic, especially farm, animals as partial human artifacts. Therefore, it is important to understand how much a particular behavior of a farm animal is far from the natural one of its ancestors. This paper is a contribution to better understand the role of genetics of the farm animals on their behavior. This means that the naïve approach to animal welfare regarding returning animals to their natural state should be challenged and that welfare assessment should be considered.

## 1. Introduction

Ethics in animal production is a hot topic and a huge amount of literature has been published [1]. One of the most debated topics in the past few decades, both at scholarly and public opinion levels, is the question of whether animals farming would be considered morally justified. A recent multisectoral contribution on ethics in animal-sourced food has been published in a monographic issue of Animal Frontiers [2]. Vegetarian and vegan movements are deboarding from their original nutritional ideological beliefs to broadly embrace the animalist party point of view, claiming that animal products, mainly meat, must be banned in human diets [3]. At the heart of the recent public debate on human–animal relationships, there is the growing awareness of citizens that the use of animals for human purposes must include the obligation on the part of farmers to guarantee them a high level of welfare [4].

It is worthy to note that the word “welfare” may assume different meanings depending on the context; the main two refer to a “physical and mental health and happiness, especially of a person” and “to help given, especially by the state or an organization, to people who need it, especially because they do not have enough money” [5]. Under an operational point of view, as needed for scientific purposes, the concept of welfare applied to farm animals has undergone a remarkable evolution until it reached a formulation that includes the following fourfold aspect: (a) biological and technical definitions, that emphasize the basic needs of animals and the freedom that should be given and the possibility of coping with environmental challenge; (b) regulation approaches, according which animals are sensitive creatures so they must be reared in the environment compatible to the biological needs of the species. This leads to the translation of the concept into a legal framework; (c) philosophical approach, discussing the role of animals in the humans’ societies; (d) interactive approach, that considers communication between farmers and animals and its impact on livestock systems [6]. 

Among biological approaches, neurocognitive studies have begun to achieve important results since Charles Darwin [7], and his student Georges Romanes [8], proposed that human mental states represent a continuum with animal ones deriving from them by evolution. Later, several scholars criticized this point of view, arguing that the animals’ traits that can be found in humans, and not the other way around. However, Darwin himself was conscious that arguing “the existence of human traits in animals rather than animal traits in human beings allowed him to express his point of view on animal and human continuity in a more acceptable way for contemporary society in which, as Elizabeth Knoll [9] observed, feelings anthropomorphic were popular in the upper middle classes” [10]. Over a century of comparative neurocognitive studies in animals and humans, which summarization is quite difficult and overtake the scope of this contribution, have led the scientific communities to converge on the belief that higher animals (and here the controversy moves to the border that demarcates this category) are endowed with mental states such as to generate internal representations of the external world. This entailed the normative evolution that established for animals a state of sentient organisms (e.g., EU Lisbon Treaty, 2007) [11], that can express basic sensations such as fear, pain and pleasure. Thus, several laws were issued to protect and safeguard their well-being. However, neurocognitive scientists have established that animals raised for economic purposes (e.g., milk and meat production) show differences in cognitive abilities and brain lateralization that can affect adaptive behavioral, physiological, and immune responses to environmental stressors [12]. However, evidence of advanced cognition in animals says little about their sentience (i.e., feeling) [13] and the lack of direct proofs, acquired only through verbal abilities, advise to consider alternative hypotheses before attributing conscious states that can account for animal behavior [10]. On the other hands, at the begin of this century a behaviorist line of thinking emerged; this new point of view has based the evaluation of animal welfare on the “emotions” of farmed livestock [14], opening a controversial field of discussion between animal sensations, feelings and emotions [15]. 

The classic research of Belyaev [16] and his students on silver foxes have shown that the domestication process, including in the specific case the selection of the animals that presented less fear of man, affects not only the morphological characters of the animals, but also (and especially) the behavioral ones. Recently, Kukakova et al. [17] have sequenced and assembled the genomes of the fox of Belyaev experiment and re-sequenced a subset of foxes from the tame, aggressive and conventional farm-bred populations to identify genomic regions associated with the response to selection for behavior: they found 103 regions with either significantly decreased heterozygosity in one of the three populations or increased divergence between the populations, demonstrating the genetic based behavior in domestic animals. 

Therefore, news perspectives could be opened for animal welfare reasoning around the concept of domestic, especially farm, animals as partial human artifacts (in this review we assume for human artifact or construct the meaning of what, in this case animal, modified by human for his own purposes). Therefore, it is important to understand how much behaviors of farm animals are far away from the natural ones of their ancestors. The consequences bring directly to assume a mixed natural and anthropocentric point of view when we discuss about farm animal welfare. A pioneering work aimed at founding a theory of the effects of domestication on animal behavior was carried out by Price [18] who, on the basis of the already abundant literature available at that time, analyzed the factors that mainly influenced the changes in their character.

This review analyses the effects of domestication and artificial selection on animal behavioral traits coupled with morphological ones. A wide literature has been considered to contribute to better understand the role of genetics of the farm animals on their behavior, finalized to demonstrate that as they are partially human artifacts, and so that their welfare should be assessed taking into account the artificial environment where farm animals have been selected. Scopus^®^ database was firstly scanned by using “bioethics, animal behavior, behavioral heritability, animal selection and behavior” as keywords; furthermore, general literature on animal ethics and animal welfare was taken into account. Results coming from the analyzed literature were extracted, classified and discussed, to derive our original points of view.

The hypothesis we want to demonstrate in this review is that the naive approach to animal welfare that brings it back to a natural state is not completely right and that welfare assessments must necessarily consider that the behaviors of farm animals have been partially, but consistently, constructed by humans.

## 2. Domestication Changed Animal Traits 

Domestication is a lively area of scientific research in which no consensus has been reached on its meaning yet. For our purpose we adopted the Zeder co-evolutive point of view [19]: she defines domestication as “a sustained multigenerational, mutual relationship in which one organism assumes a significant degree of influence over the reproduction and care of another organism in order to secure a more predictable supply of a resource of interest, and through which the partner organism gains advantage over individuals that remain outside this relationship, thereby benefitting and often increasing the fitness of both the domesticator and the target domesticate”. Under this framework, Zeder [19] observed that human domestications are different from non-human ones because we have been able to opportunistically grasp and modify the traits that have increased the benefits obtained from co-evolutionary relationships with the target species. Moreover, we have transmitted these practices capable of achieving our goals not only within the parental circle, but also to all through social learning. Animal domestication is a cultural issue of Homo sapiens because, until now, no trace of similar practices has been found by archaeologists for the most ancient *Hominidae* species, even though they have been in continuous contact, as hunters, with wild animals for millions of years.

Restricting this field to livestock, when humans became sedentary farmers, they started to domesticate wild animals to fit different needs, such as work, and meat or wool production. This can be considered at all the first step of the artificial selection processes. However, during this long time of human–animal partnership, natural selection has continued to shape the domestic animals but, as Darwin [20] firstly observed, these factors have been overwhelmed by the artificial ones. For this reason, in this review we have considered the effects of human selection only. Beside historical and archeological traces, modern genomics tools allowed to study and define a timeline of the domestication process [21,22,23]. The domestication of livestock species started in Southwest Asia (the Near East) about 10,000 years ago with goats, sheep, humpless cattle, pigs and then water buffalo, horse, chicken and, more recently, rabbit [24,25,26]. Horses were domesticated for their key role in warfare and transportation during multiple events somewhere in the Eurasian plains [27]. An interesting case is the rabbit (*Oryctolagus cuniculus*) which is a key species (is the only relevant mammal livestock species originating from Europe at the Roman empire age) because of its role as livestock, game, pet, and experimental animal (as well as pest in several countries, mainly Australia) [28,29].

Domestication process changes phenotypic and behavioral characteristics of wild animals depending on the species. Behavioral and morphological traits of modern livestock breeds resulted from artificial selection of natural useful characters: in fact, domestication process increased the phenotypic and genetic variability of breeds and the modern biodiversity is one the most important outcomes of the domestication before and selection later. Under this point of view, the domestication created prototypes of each species that were subsequently shaped by the artificial selection process leading to an increase in the variability, in terms of both genetic and phenotypic richness. Rasali et al. [30] reported that humans all around the world raise about 200 and 400 pure and composite sheep breeds, respectively. Even if the exact knowledge about the sheep descendant is still debated, all these breeds, characterized by different morphological traits and ability to adapt to extremely different environments, seem to be originated from just a couple of wild ancestors. Let us think also to bovine (Bos taurus) and zebuine (Bos taurus indicus) breeds that, even showing important differences between and within them, share the same wild ancestor, the aurochs (Bos primigenius): starting from this, more than 1000 cattle breeds are nowadays raised worldwide [31]. Of particular interest is also the pig of which 566 different breeds can be enumerated, all originating from the wild boar [32]. Domesticated sheep and goats show different features compare to their wild ancestors, such as reduced body size and horn length [23,33]. Domesticated cattle breeds show smaller size compared to the extinct wild aurochs and they developed the capacity to adapt to various environments [34,35,36]. However, some cattle breeds have longer horns with particular shape that were selected for religious reasons [27]. Another interesting phenotype shaped during domestication process is the coat color, that in some species (such as horse, cattle and sheep) is a useful trait to discriminate among breeds.

Domestication changed, not only external features, but also behavior and aptitude of livestock species [18,37]. For example, sheep and goats were chosen for their meekness and for their multiple productions (e.g., meat, milk, wool and horns). However, the mechanisms behind this process were not still completely clear, and different theories have been proposed during time, until second half of the twentieth century, when the modern concept of ethology raised [38]. The behavioralists of the past believed that changes in animals’ behavior were mainly due to the different environments, learning and nurture. Nowadays, this concept has been abandoned in favor of a more realistic one involving a genetic determinism [39,40,41]. This is particularly true because a trait without heritable mechanisms could not play a role in the evolution process. Other authors proposed a theory, in which neural crest cells play a key role, called “domestication syndrome” to put together all the differences in phenotypic, and therefore genetic, characteristic between wild and domesticated species [42,43]. The term domestication syndrome applied to animals came from the domesticated crop plants [44]. As specified by Sánchez-Villagra et al. [45], the word “syndrome” is not related to a particular pathological condition or disease, but it came from the literature about plants that humans selected for interrelated syndromes of characteristics during the domestication process [46]. Sánchez-Villagra et al. [45] reported that some modifications are common to almost all domesticated animal species: increased docility, increased skillfulness in using human cues, increased fecundity, reduction of tooth, brain and rostrum size, floppiness of the ears, curliness of the tail and depigmentation of skin and fur. Two main hypotheses were suggested for explaining the differences between wild and domesticated species: the first one state that these differences are due to the new environment in which domesticated animals received improved diets and found better live conditions. The second one suggested that domestication syndrome was related to hybridization between different breeds or even species. Anyway, livestock species undergone domestication syndrome show differences in morphological and behavioral traits compared to their presumed wild ancestors. (Table 1).

Regarding the changes in animals’ behavior, all features of farm animals that are currently raised by humans show heritable genetic variations [47,48]. Fear and interactions with humans, sexual maturity and social behavior were also affected by domestication. Farm animals show less fear of humans, and they are more inclined to cooperate with humans with a lower stress level. Farmed pigs reach sexual maturity at about 6–7 months, whereas for wild boars, sexual maturity is reached at approximately two years [49]. Moreover, farm animals are forced to live in larger groups, compared to their wild ancestor, under crowded conditions [48]. York [50] reported a list of the behavioral traits, and the tests used to study them, that have been analyzed in livestock species.

Genomic studies allowed to trace the domestication process and to highlight genomic regions where genes involved in the phenotypic differences among breeds are mapped. For example, Cesarani et al. [51] compared two related sheep breeds, one showing ancestral traits and one selected for milk aptitude, and they found significant genomic regions that harbor gene involved in morphological traits such as coat color (MC1R, MITF), horned/polled (RXFP2) and body size (NPY, VIP). Moreover, the authors identified the HOXB1 gene that was already associated with the *anotia* phenotype in mice [52], that is of interest because the breed with ancestral traits is characterized by microtia, external malformation trait that is often present in wild animals [53,54]. Hornedness/polledness is one of the main traits modified by human selection during the domestication from wild ancestor to modern breeds [55]: e.g., ancestral wild sheep showed big horns useful as defense and injury instrument.

Several studies focused their attention to the different genetic background (e.g., different gene expression) between wild and domesticated animals [56,57]. One of the ideas is that these differences are not related to different environment and methods of farming, but they are heritable traits that can be transmitted to the progeny [58,59,60]. This theory seems to be in contrast with the first one of Charles Darwin [20] in which the theory of heredity was absent. Domestication is one of the most important events in mankind’s history, which has now consequences that are intrinsic in the everyday reality. Recently, some studies highlighted that species undergone domestication for thousands of years, showed changes in behavior, color, morphology and physiology which are related to several independent genomic regions [61,62]. However, studies on species with more recent domesticated history (e.g., cat and salmon) revealed fewer signals [63,64] compared to the older domesticated species. Thus, these changes could be associated with the extension of the domestication process. This hypothesis is strengthened by the fact that a long time is needed to fix the heritable traits, especially those with lower heritability; even under strong selection pressure, only small changes in gene frequencies can be observed over several generations [65]. On the contrary, recent investigations demonstrated that sometimes only few [66] or exceptionally just one [67] generations could be enough to show genetic adaptation to captivity. In order to test this idea, Christie et al. [60] analyzed gene expression between the offspring of first-generation hatchery trout and wild trout reared in an identical environment. The authors found 723 genes differently expressed in the two groups of animals, mostly involved in responses in wound healing, immunity and metabolism.

As aforementioned, the genomic studies were able to identify selection signatures, i.e., genome regions shaped by selection, that can differentiate wild and domesticated animals. In these regions, genes encoding for the phenotypic differences were mapped. Due to involvement of genes and the fact that progenies of wild and domesticated animals show feature like their ancestor and not to the opposite group, genomic mechanisms of the domestication, and therefore its heritable pathway, could be confirmed. 

## 3. Questions and Answers about Modern Farm Animals

Domestication, firstly, and genetic selection subsequently, are shown to be effective tools to change the temperament of farm animals orienting it towards the desired behavioral characteristics (e.g., docility, resistance to adversity, patience, maternal attitude…). Current characters of farm animals were demonstrated to be a human construct, and that they can be further quite rapidly oriented by their insertion among the breeding goals of genetic selection. Nowadays, we are faced with several ethical questions, and subsequently approaches to assess and improve animal welfare. 

Ethic is a philosophical rich field that explores the unknown answer by dealing originally with known arguments. According to Floridi [68], philosophers usually ask open questions to which they usually do not provide definitive answers. However, they tend to define the problem space and within it they try to give the most probable answer according to the state of knowledge. Under the recent constructivism point of view, the philosopher is a model builder who reduces the problem space at the minimum manageable to further connect these elements in a most complex and complete framework. In this way, the scientist is a special type of philosopher who deals exclusively with real facts instead of abstract entities, for which however he seeks an answer based on experience and continually revisable according to the evidence of new elements [69].

Question one: Does the character change in domesticated animals by human purposes shifted some of the responsibility for their survival and well-being into human actions?

As domestication is a particular case of coevolution between living organisms, the pervasiveness of humans should modify the environment and consequently the factors influencing the behavior in wild animals also. In fact, animals living near humans (e.g., in contexts like captivity and urbanization) rapidly change their antipredator behavior and become tolerant to people’s presence [70]. This justifies the growing awareness to take care for wild animals (e.g., care in wildlife centers in case of disease, foraging in case of food scarcity, etc.), even if this can divert their evolutionary trajectory because of the manipulation of the natural fitness factors. While it is generally accepted that humans are responsible for the welfare of wild animals by manipulating part of the natural environmental factors to protect them, the share of human responsibility on the welfare of farm animals is greater due to the fact that some behaviors have changed due to artificial selection. This means that the breeders, when designing their welfare programs, must consider the distance that exists between the behavioral construct of the animals they reared and that of the natural environment, paying greater attention to the needs of the animals, the greater this distance. In other words, high productivity animals kept in more intensive farming systems have a greater need for attention to their welfare than less productive ones reared in extensive systems. Just a few examples are the superior care associated with intensive systems, such as higher veterinary care, control of the production environment in terms of temperature, humidity and light, and better feeding and managements strategies [71]. Another example is represented by the use of piglet lamps in the sow farrowing pens compared to body heat of the mother in extensive pig systems [72]. It follows that animal welfare is a technology that, like the others used for animal breeding, must be studied and applied not only for ethical purposes, but also to obtain the best coupling between available environmental conditions and animal health and productivity.

Question two: Can the behavior be indicated as genetic goal in animal selection? The starting point of this answer must necessarily rest on the principle, initially elaborated by Rollin [73] and subsequently perfected by Shriver [74], of the conservation of welfare in animals subjected to genetic manipulation: “any animals that are genetically modified through the use of genetic technology [for our intent, this means both by classical genetic selection of genetic manipulation], for purposes other than research, should be no worse off, in terms of suffering, than the parent stock was prior to genetic alterations.” Several genetic studies demonstrated that behavioral traits are inheritable: estimates mostly range from 10 to 50% [47,50,75,76,77]. The interest in estimate heritability of behavioral traits of farm livestock lies in the past: Kjaer and Sørensen [78] estimated the heritabilities of cannibalism and feather pecking in laying hens of White Leghorn breed. In fact, restricted and crowded spaces bring the hens to attack other animals or even injury themselves and farmers try to prevent these behaviors through beak trimming, practice under scrutiny by animals’ right associations [79]. Due to these heritable mechanisms, behavioral traits can become (and sometimes are already) the goal of breeding scheme. 

As aforementioned, also behavior was shaped by the domestication process and therefore genetic studies analyzed also this range of traits. For example, Ding et al. [80] found a single intronic retroelement involved with variations in courtship dance in Drosophila species. York [50] analyzed more than one thousand genomic loci (of mammals, birds, fish, insects and nematodes) associated with a series of behavioral traits (such as: courtship, feeding, aggression, motor, emotion, temperament, learning, social, parental and circadian) and he confirmed the genetic determinism of such traits. This author reported that courtship and feeding behaviors are determined by genomic regions of significantly greater effect than other traits. Boissy et al. [81] analyzed the effect of genotypes (i.e., different breed composition) of both lamb and dams on the emotional reactivity in sheep. These authors concluded that most of the differences were due to direct additive genetic effects and that females were more active and avoided more the human contact compared to male lambs.

Reduce the aggressivity of animals would have a series of positive benefits both for animals and humans. Let us focus on pigs and on the positive benefits in this species. Turner et al. [82] investigated the genetic aspects of individual aggressiveness in pigs by the association of skin lesions and behavioral traits. Animals living in crowded situations tend to be more aggressive, even if aggressivity to get access to resources can be also found in nature (for example regrouping at pasture) to define a new social order [83,84]; in pigs there are both reciprocal and non-reciprocated aggressions. In both cases, animals suffering these assaults are more stressed, reduce their feed intake and therefore their productions. One of the main problems related to behavior in pigs is the aggressive behavior of sows towards their piglets: this problem has also been largely studied since the past. Already in the late twentieth century, Knap and Merks [85] analyzed the aggressiveness of primiparous sows and reported a decrease of litter size at weaning of almost two piglets per litter. These authors reported heritabilities ranging between 0.40 and 0.90. These numbers show the effectiveness of including the mildness among the breeding goals: through genetic (or now genomic) selection, the aggressiveness of livestock animals can be reduced by having positive effects in terms of animal welfare and production.

Another behavioral trait is the fighting aptitude in South America and European cattle breeds [86]. One example is the Spanish fighting bull (known also as Iberian or Lidia bulls) which is raised in Spain, Portugal, France and South American countries (e.g., Mexico) for bull fighting events [87,88]. Another example are the not cruel traditional events of fighting among cows belonging to Aosta Chestnut and Aosta Black Pied [89] or Valdostana [90,91,92] cattle breeds in North Italy (Alpine regions). Both tourism and farmers take important economic advantages from this kind of contest, named “battle of queens”. At the end of October, pregnant cows are divided in classes according to their size and they fight (by pushing forward the other animal using their weights) without injuring the opponents. The cow winning this tournament becomes the “queen of all queens” and it assures huge incomes to its farmer, who can sell her or her offspring. Fighting aptitude is evaluated and breeding values are estimated for Valdostana Pezzata Nera and Castana cattle breeds; this behavioral trait is already included in the breeding schemes of these breeds.

Further important “behavioral” traits shifted by selection are adaptation and resilience [93]. The animal production is now become more efficient and, therefore, each unit of production shows a smaller environmental footprint [94]. Moreover, animals are not only more efficient, but they adapted to produce in very different environments. However, areas of production are likely going to become harsher (e.g., increased heat, disease challenges) [93]. Thus, if we consider the heat-tolerance among the “behavioral” traits, adaptability to difficult environments is (and will be) a strategic point. Resilience is mostly defined as the capacity to recover quickly from difficulties. Unfortunately, it seems at resilience is negatively correlated with genetic selection pointing to increase production traits, which reduced environmental adaptation [93]. Frankham [95] stated that genetic selection for the adaptation to captivity reduced fitness, which is confirmed when animals are reintroduced into the wild. Thus, selection for resilience will largely acts on morbidity, longevity and mortality rather than productive performance. Resilient animals may show lower performances in optimal environments [93]. 

It is interesting to note that the more recent domestication of rabbits has made it possible to retain many of the ancestral and wild behaviors, like living in small groups of 2–8 individuals with familiar subgroups, spend the time mainly underground and coming outside only for foraging, stand up to check for any predators, locate a specific area for the true feces (scrapes), etc. When these animals are placed in free-range conditions, they make nests and dig burrows [96].

Finally, two other species in which behavior has a large impact are dogs [97,98], and horses [99,100]. It is worthy to mention these species because of their tight relationship with humans: dogs and horses are raised as companion (both), working (mainly dogs) or sport (mainly horses) animals. Thus, it is compulsory that these animals show low aggressivity towards humans. Years of selection of candidates based not only on performances, but also on conduct, led to animals with tame behavior particularly suitable for staying with children or disabled: just think of dogs for blind people or horses for therapeutic riding. Without selection for behavioral traits, these animals improving the life quality of humans there would not have been.

Question three: How can farmers achieve the highest possible welfare for their animals? The sustainable intensification of livestock chains is the narrow way to simultaneously meet the growing demand for food of animal origin and the reduction of their environmental and social impacts [101]. The economic pressure to reduce costs for maintaining the viability of farming enterprises and the scaling order that assigns more animals per operator, have diverted the perspective in the design and construction of farming environments towards savings and removed the animals from the direct control of the farmers. Furthermore, the need for manpower on farms with the increasing insert of employees of non-rural extraction or coming from work paths other than breeding, has led to the drop in tension for animal welfare based on life experience or on work or training courses adequate to the ethical challenge in game. Finally, over a century of selection based on functional traits has placed character traits in the background, with the consequence that technologies have to chase the well-being of current animals as their behavior has not been subject to selection as had happened since the dawn of domestication. 

To address sustainable animal welfare—where the word “sustainable” means a reliable and enduring set of knowledge developed on ethical and economic assumptions applied to livestock farming—the environment (physical and physio-phycological) where the animals will spend their lives must be designed and built with primary welfare in mind reachable by the real subjects (i.e., by the concrete individuals that will inhabit the precise temporal-physical space). Precision livestock farming (PLF), one of the information and communication technologies (ICT) often installed at farm level with no consistency and underutilized by farmers, needs to be designed and geared consistently to reduce stressors and to monitor on an individual level each animal, replacing the eyes and expertise once owned by the breeders.

An attempt to adapt breeding environments to the high performances of current animals is those pursued by the Ethical Barn^®^, a new way to treat and stay dairy cows in balance between social sustainability (farmer’s income), environmental sustainability and respect for the right of the cow, aiming to give a decent life to cows [102]. This holistic approach allows to manage dairy cows with very high production performances—as the 20 tons/head of milk per lactation indicated as phenotypic to achieve in 10 years by the top dairy farms—while minimizing physical and mental stress, sources of infection and environment impact. 

Unlike ruminants, in which the extra-resting circadian time budget is mainly spent on feeding-rumination-milking/suckling activities, in monogastric the conditions of captive breeding involve large time spaces that must be filled to avoid alienation phenomena and consequent psychological discomforts. In pigs, environmental enrichment is a rapidly developing technique, also supported by regulatory obligations within the EU [103], but which faces many obstacles globally to be applied correctly [104].

The Info-Farming revolution is under our eyes, supported by the evolution of the PLF, which allows the rapid diffusion of sensor devices with greater process data capacity at lower costs and pervasive ITC, which have become a prevalent part of our life today. In this last aspect, the spread of smartphones capable of collecting real-life snapshots and delivering them to the social media circuit in real time represents a challenge to the adoption of correct animal welfare procedures on farms. At the same time, the creation of social communities among breeders makes possible to exchange technical information and to share welfare procedures capable of rapidly improving the living conditions of animals raised on their farms. 

One of the most important aspects is to bring the animals closer to the breeder, especially when large numbers of animals and large breeding facilities prevent real control of the animal welfare conditions [105]. As pointed out by Neethirajan [106], artificial intelligence (AI) can represent the next step to reach the better animal outcomes, processing and making strategic decisions to improve the welfare and health of farmed animals.

**Figure 1:**
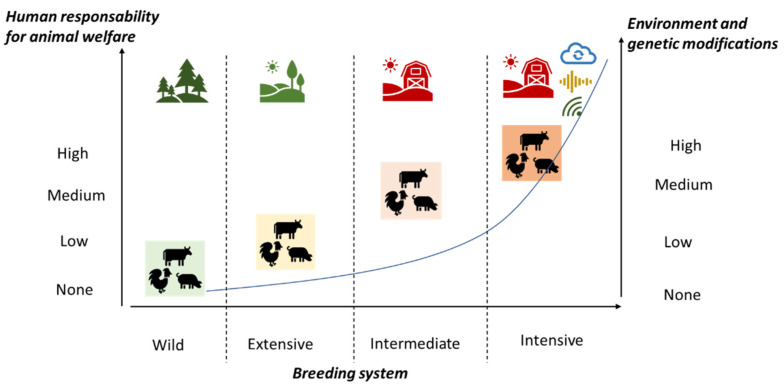
Human responsibility for the welfare of farm animals increases as the genetic modification of livestock and the technological evolution of the farm environment increase.

Summarizing, the profound changes in animal genetic and breeding environments mean that farmers are increasingly committed to livestock welfare. This goal can be achieved both with an adequate genetic improvement, which also has as its goal the behavioral characteristics associated with the well-being of animals in modern breeding conditions (genetics for environmental adaptation), and with the massive use of PLF technologies capable to meet the growing needs of increasingly performing animals (environment for high-producing animals) (Figure 1).

## 4. Practical Implications

Current farm animals are the result of millennia of selection which has led to their departure from the physiology and behavior of their ancestors. Cows that produce 12,000 L of milk, steers that grow by 2 kg per day, sows that give birth to 30 piglets per year and hens that lay 280 eggs per year are phenotypes very far from the performance of their wild ancestors (e.g., 1000 L of milk per year per cow, 0.3 kg/d of body weight increase in steers, 8 piglets/year per sow and 20–25 eggs per year per hen) which were commensurate with the turnover rate of the natural population in balance with its environment. The one to two order of magnitude of biological surplus produced by the modern livestock, used for human food interests, places these animals under great metabolic effort which affects their physiological and psychological well-being. With increasing breeding intensity, which is necessary to obtain higher yields, the environment and farm routines are profoundly changing. This means that farmers in intensive systems must pay more attention and make more investments to ensure the welfare of their animals. Farm animals are partially human-made artifacts that bear the responsibilities related to the metabolic fatigue they endure to render the important service of producing high quality food for humanity. Their welfare must be guaranteed in all breeding conditions and in all countries of the world.

In practical terms, current breeders have to deal with an increasing complexity in managing farms and with the containment of production costs. The design of comfortable environments suitable for high production needs is the first requirement for animal welfare, followed by respect for the management of the groups and the circadian needs of the various categories. Highly selected animals have sophisticated dietary needs not only in quantitative terms, but above all in the way they eat their food. Breeders must take into account specific feeding behaviors and schedule and program schedules and rations to meet the specific needs of their animals. High production also implies greater exposure to diseases for which farmers must adopt the best practices suggested by international organizations [107]. Finally, the complexity of managing a modern livestock farm requires continuous training of the staff and the careful vigilance of the entrepreneur so that all legal and company regulations are respected. It is worthy to note that animal welfare certification is recently wide spreading throughout the implementation of the Welfare Quality (WQ) protocols (based on the effects of the farming system on the animal condition-welfare), due to the fact that animal welfare must be guaranteed in all breeding conditions and that people in general, and consumers of animal products in particular, are interested in being sure that products they consume come from animals that have been correctly reared and managed.

## 5. Conclusions

This review confirms the hypothesis that the behavior of highly selected farm animals is long away from its natural state. There are behavioral changes in some farm animals compared to their wild ancestors due to genetic selection-domestication which should be considered in planning and implementing farm animal welfare standards at the farm level and an upgrade in the regulatory body.

Animal scientists must push governments to ensure that the welfare standards required by the regulations are universal and guide the international trade in products of animal origin; at the same time, they must devote more and more research spaces to make available scientific elements and technologies capable of making this reality operational and at acceptable costs everywhere.

## Figures and Tables

**Table 1:** Morphological and behavioral traits associated with the domestication syndrome that changed from wild ancestor to domestica livestock species (adapted from Wilkins [42]).

Trait	Livestock Species
Curly tails	Dog, pig
Depigmentation	Cattle, dog, goat, horse, pig, rabbit
Docility	Cattle, dog, donkey, goat, horse, pig, rabbit, sheep
Floppy ears	Cattle, donkey, dog, rabbit
More frequent estrous cycles	Dog, goat
Neotenous (juvenile) behavior	Dog
Reduced ears	Dog
Shorter muzzles	Cattle, dog, goat, pig, sheep
Smaller brain or cranial capacity	Cattle, dog, goat, horse, pig, rabbit
Smaller teeth	Dog, pig

## Data Availability

This paper did not involve any dataset.
